# Potential contribution of prescription practices to the emergence and spread of chloroquine resistance in south-west Nigeria: caution in the use of artemisinin combination therapy

**DOI:** 10.1186/1475-2875-8-313

**Published:** 2009-12-30

**Authors:** Grace O Gbotosho, Christian T Happi, Abideen Ganiyu, Olumide A Ogundahunsi, Akin Sowunmi, Ayoade M Oduola

**Affiliations:** 1 Malaria Research Laboratories, Institute of Advanced Medical Research and Training, College of Medicine, University of Ibadan, Ibadan, Nigeria; 2 Department of Pharmacology and Therapeutics, College of Medicine, University of Ibadan, Ibadan, Nigeria; 3 Empowering health researchers, Tropical Disease Research (TDR), World Health Organization Geneva, Switzerland; 4 Stewardship for Research, Tropical Disease Research (TDR), World Health Organization Geneva, Switzerland

## Abstract

**Background:**

Prescription practices have been shown to influence the emergence of anti-malarial drug resistance. Thus efforts in this study were devoted to evaluating the prescribing practices prior to introduction of the artemisinin based combination therapy (ACT) in Nigeria and its potential contribution to emergence of chloroquine resistant malaria in south-west Nigeria, in order to forestall a similar situation with the ACT.

**Methods:**

A retrospective quantitative study was designed to examine case records of patients treated for malaria in either a government or a private hospital in Ibadan, south-west Nigeria, over a 20-year period, cutting across three phases of resistance to chloroquine in Nigeria: pre-resistance, emerging resistance and dissemination of resistance. Patient prescriptions were examined for use of anti-malarial drugs, sub-therapeutic doses of chloroquine, co-administration of anti-histamines with chloroquine. Descriptive statistics of frequency and percentage were used to describe trends in the parameters assessed using EPI-info.

**Results:**

Case record files of 2,529 patients were examined. Chloroquine was the main drug used in treatment of malaria throughout the periods studied, with frequency of prescription at both sites ranging from 91.4% to 98.3% during the pre-resistance years. It was administered as standard doses during the pre resistance years. Anti-histamines, especially promethazine, were routinely co-administered with chloroquine at this period too. However, the practice of prescribing sub-therapeutic doses of chloroquine at the private health care facility coincided with the latter phase of emerging resistance and phase of dissemination of resistance. Frequency of prescription of sub-therapeutic doses increased from 6.7% in 1983 (pre-resistance years) to 43.6% in 1997 (dissemination of resistance phase) at the private health care facility. Frequency of co-administration of anti-histamines with chloroquine also reduced during the period of dissemination of resistance.

**Conclusion:**

The results from this study describe a lack of adherence to national treatment guidelines, especially in the private sector, and a relationship between prescription practices and dissemination of drug resistant falciparum malaria. As Nigeria adopts the use of ACT, there is an urgent need to improve malaria treatment practices in Nigeria in order to prolong the clinical shelf-life of the combination.

## Background

The change in malaria control policy in Nigeria in 2005 in favour of artemisinin-based combination therapy (ACT) became necessary with the prevalence of *Plasmodium falciparum *resistance to chloroquine and sulphadoxine-pyrimethamine [1-6]. Prescription practices have been shown to influence the emergence of resistance to anti-malarial drugs [7,8], thus the success of a new treatment policy would depend on the adherence of health providers and patients to treatment recommendations [9]. This becomes important in order to protect the clinical shelf-life of the artemisinin-based combinations since they remain the most valuable drugs currently available for the management of malaria. Irrational use of ACT could undermine one of the goals of combination therapy, which is to prevent the emergence of resistant malaria parasites. Drug use patterns can be evaluated in terms of prescribing and dispensing practices as well as patients' use of the drug. The private sector has been shown to be responsible for treating half of the malaria cases in Nigeria [10] and the role of prescription practices in the emergence of anti-malarial drug resistance in Nigeria has not been fully elucidated.

This study focused on evaluating the prescribing practices of medical practitioners in public and private hospitals prior to introduction of ACT in Nigeria and the potential contribution of the prescribing practices to the emergence of chloroquine resistant malaria in south-west Nigeria, in order to forestall a repeat of this with ACT. It is hoped that the study would provide valuable guidelines on the use of ACT in Nigeria in order to prolong the clinical utility of the drug. A retrospective study was designed to examine case records of patients treated for malaria in two major government-owned and private hospitals in Ibadan over a 20-year period.

## Methods

Ethical clearance was obtained from the joint University of Ibadan/University College Hospital Ethical Committee.

### Study site

The retrospective study was conducted in Ibadan, south-west Nigeria. Malaria is hyper-endemic in the area with perennial transmission. The climate is that of tropical rainforest zone, with a warm dry-season from November to April and a rainy season from May to October. The study was originally designed to evaluate patient case records at three government-owned hospitals and private clinics in Ibadan, but most of the hospitals lacked the records required for the study or prevented access to the records. The study was thus conducted at only two sites, a government-owned hospital and a major private hospital in Ibadan, with an evaluation of case records files from the male, female and children's wards.

### Data collection

The data were collected in 2001. The retrospective study on prescription pattern was conducted across three phases of resistance to chloroquine in Nigeria; pre-resistance phase (1980-1986), phase of emerging resistance (1987-1993) and phase of dissemination of resistance (1994-2000). The data from case record files were evaluated if the following information were available: diagnosis for malaria, prescription of anti-malarial drugs, indication of dose administered and duration of therapy, patient medical history, especially previous use of anti-malarial drugs prior to hospital visit. The following information were obtained from the case record files: the number of times of prescription of each anti-malarial drug (chloroquine, sulphadoxine-pyrimethamine, quinine, amodiaquine, mefloquine or halofanthrine) per annum, the number of times of prescription of sub-therapeutic doses of chloroquine (< 25 mg/kg body weight) across the selected period, rate of co-administration of resistance modulating agents such as antihistamines or calcium channel antagonists with chloroquine, relative use of various types of resistance modulators co-administered with chloroquine and the number of times of co-administration of other drugs with anti-malarial properties (co-trimoxazole, antibiotics). Descriptive statistics of frequency and percentage were used to describe trends in the parameters assessed using EPI-info. Chi-square tests were used to compare proportions of appropriate prescriptions over the years. Trend of development of resistance to chloroquine in Nigeria during the period studied was obtained from published studies.

## Results

A total of 2,190 patient case notes were evaluated at the private hospital during the study of prescription pattern and at least 300 case notes were evaluated for each of the selected years. A total of 339 case notes were evaluated at the Government owned hospital (being a referral centre for complicated cases). Malaria was diagnosed in all the patients by microscopic examination of blood films in addition to clinical signs and symptoms of malaria.

### Prescription of anti-malarial drugs

In the pre-resistance years (1980-1986) chloroquine (CQ) was the most commonly prescribed anti-malarial drug accounting for 94.8% to 97.4% of anti-malarial drug prescription at the private hospital between 1983 and 1985 (Table 1). Other anti-malarial drugs prescribed during this period included sulphadoxine-pyrimethamine (SP) and amodiaquine (AQ) (frequency of prescription was 2.4% and 2.8% respectively). A progressive reduction in prescription of CQ through the phases of emerging resistance (1987-1989) and the resistance dissemination phase (1990-1997) was observed. The frequency of prescription of SP and AQ increased to 4.1% and 24.7% respectively in 1989. Quinine (QN) was also used during this period, but frequency of prescription was very low (0.3%). By 1997, frequency of prescription of SP had increased to 13.4%. Other anti-malarial drugs prescribed during this period included halofanthrine and artemether as monotherapy (frequency of prescription was 3.1% and 1.6% respectively) (Table 1).

**Table 1:** Frequency of prescription of anti-malarial drugs at a private hospital in Ibadan, south-west Nigeria, over a twenty-year period.

YEAR	CQ	SP	AQ	QN	PY	PG	HAL	ART
1983 N = 303	94.8%	2.3%	2.8%	0	0.3%	0	0	0

1985 N = 306	97.4%	0.7%	1.0%	0	0.3%	0	0	0

1987 N = 304	95.5%	1.9%	0.6%	0	1.0%	1.0%	0	0

1989 N = 320	67.1%	4.1%	24.7%	0.3%	0.9%	1.3%	0	0

1992 N = 322	80.1%	10.2%	0.6%	0.9%	0.3%	1.2%	5.9%	0

1995 N = 314	77.4%	10.2%	4.1%	1.9%	0.6%	1.9%	3.8%	0

1997 N = 321	73.7%	13.4%	2.8%	2.2%	0	1.9%	3.1%	1.6%

CQ = Chloroquine; SP = Sulphadoxine/Pyrimethamine; AQ = Amodiaquine; QN = Quinine; PY = Pyrimethamine; PG = Proguanil; HAL = Halofantrine; ART = Artemether

Similarly, in the government-owned hospital, CQ was the most commonly prescribed anti-malarial drug during the pre-resistance years. Amodiaquine was the only other anti-malarial drug prescribed during the pre-resistance years. However, frequency of prescription of CQ at the hospital reduced from 98.3% in 1983 to 80.7% in 1989 and 68.4% in 1995 during the phases of emerging resistance and resistance dissemination respectively (Table 2). In 1989 the frequency of use of AQ and QN were 10.5% and 3.5% respectively. Between 1992 and 1997 the frequency of use of sulphadoxine-pyrimethamine varied between 8.8% and 15.8% while use of quinine increased to 10.5% in 1995. Halofanthrine and artemether were also prescribed between 1992 and 1997 though the frequency of prescription was low (1.7% to 1.8%) (Table 2).

**Table 2:** Variation in prescription of anti-malarial drugs at a government-owned hospital in Ibadan, southwest Nigeria over a twenty-year period.

Year	CQ	SP	AQ	QN	PY	PG	HAL	ART
1983 N = 57	98.3%	0	0	0	0	1.7%	0	0

1985 N = 58	91.4%	0	1.7%	0	6.9%	0	0	0

1987 N = 58	89.7%	0	0	0	10.3%	0	0	0

1989 N = 57	80.7%	0	10.5%	3.5%	3.5%	1.8%	0	0

1992 N = 57	86%	12.3%	0	0	0	0	1.7%	0

1995 N = 57	68.4%	15.8%	0	10.5%	1.8%	0	1.8%	1.8%

1997 N = 57	82.5%	8.8%	0	7.0%	0	0	0	1.7%

CQ = Chloroquine; SP = Sulphadoxine/Pyrimethamine; AQ = Amodiaquine; QN = Quinine; PY = Pyrimethamine; PG = Proguanil; HAL = Halofantrine; ART = Artemether

### Usage of sub-therapeutic doses of chloroquine

In the private hospital, frequency of prescription of sub-therapeutic dose regimen of chloroquine was low during the pre-resistance phase and varied between 1.7% and 6.9% (Table 3). However during the resistance dissemination phase (1994-2000) an increase in frequency of prescription of sub-therapeutic doses of CQ varying between 27.2% and 43.6% was observed (Table 3). Of the 587 patient records observed in the pre-resistance years 562 (95.7%) patients received full regimen of chloroquine compared with 25 (4.3%) that received sub-therapeutic doses of CQ. However, in the dissemination of resistance phase, 313/484 patients (64.6%) received standard doses of CQ while 171/484 (35.3%) patients received sub-therapeutic doses of CQ. These proportions were significantly different X^2 ^= 233.03, P < 0.0001. At the Government owned hospital, frequency of prescription of sub-therapeutic doses of chloroquine varied between 5.4% and 5.7% during the pre-resistance phase (Table 4) and remained low (0-7.7%) throughout the periods of emerging or dissemination of resistance (X^2 ^= 1.72, P = 0.189).

**Table 3:** Frequency of prescription of complete therapeutic regimen or sub-therapeutic regimen of chloroquine over a twenty-year period at a private hospital in Ibadan, south-west Nigeria

	Full course regimen		Sub-therapeutic regimen		
	
YEAR	N	F	N	F	
1983 (N = 289)	269	93.1%	20	6.9%	Pre-resistance phase
	
1985 (N = 298)	293	98.3%	5	1.7%	

1987 (N = 295)	284	96.3%	11	3.7%	Phase of emerging resistance
	
1989 (N = 220)	214	97.3%	6	2.7%	
	
1992 (N = 258)	202	78.3%	56	21.7%	

1995 (N = 243)	177	72.8%	66	27.2%	Phase of resistance dissemination
	
1997 (N = 241)	136	56.4%	105	43.6%	

N = No. of prescriptions evaluated

F = Frequency of prescription

**Table 4:** Frequency of prescription of complete therapeutic regimen or sub-therapeutic regimen of chloroquine at a government-owned hospital in Ibadan, southwest Nigeria over a twenty-year period.

	Full course regimen		Sub-therapeutic regimen		
**Year**	**N**	**F**	**N**	**F**	

1983 (n = 56)	53	95%	3	5.4%	Pre-resistance phase
	
1985 (n53)	50	94.3%	3	5.7%	

1987 (n = 52)	52	100%	0	0	Phase of emerging resistance
	
1989 (n = 46)	46	100%	0	0	
	
1992 (n = 49)	47	95.9%	2	4.1%	

1995 (n = 39)	36	92.3%	3	7.7%	Phase of resistance dissemination
	
1997 (n = 47)	47	100%	0	0	

N = No. of prescriptions evaluated

F = Frequency of prescription

### Co-administration of chloroquine with adjunct drugs

Chloroquine was routinely co-administered with various antihistamines or antiemetic including promethazine, chlorpheniramine, cyproheptadine, diphenhydramine or chlorpromazine during the period studied at the private hospital (Table 5). Frequency of prescription of promethazine was highest throughout the phases studied. During the pre-resistance phase, frequency of prescription of promethazine ranged from 74% to 76% while that of chlorpheniramine ranged between 5% to 6.5%. A significant decline in frequency of prescription of promethazine was observed during the phases of emerging resistance and dissemination of resistance (X^2 ^= 83.04, P < 0.0001), while frequency of prescription of chlorpheniramine significantly increased during these periods to 34% to 47% (P < 0.0001).

**Table 5:** Antihistamines co-administered with chloroquine over a twenty-year period at a private hospital in Ibadan, south-west Nigeria

	Promethazine		Chlorpheniramine		Cyproheptadine		Others	
**YEAR**	**N**	**F**	**N**	**F**	**N**	**F**	**N**	**F**

**1983**	170	74%	11	5%	3	3%	45	20%

**1985**	209	75.7%	18	6.5%	1	0.4%	48	17.4%

**1987**	181	63.7%	39	13.7%	4	1.4%	60	21.1%

**1989**	86	49.1%	70	40%	0	0	19	10.9%

**1992**	103	52.0%	75	37.8%	0	0	20	10.1%

**1995**	51	43.2%	55	46.6	3	2.5%	15	12.7%

**1997**	62	44.3%	48	34.3%	8	5.7%	22	15.7%

N = No. of prescriptions evaluated

F = Frequency of prescription

At the government-owned hospital, anti-histamines or anti-emetics, including promethazine, chlorpheniramine, diphenhydramine, chlorpromazine, cyproheptadine and pizotifen, were also routinely prescribed with chloroquine (Table 6). Promethazine was most frequently co-administered with chloroquine until 1997 when chlorpheniramine became the most frequently co-administered anti-histamine. Anti-histamines were routinely prescribed with chloroquine throughout the period studied at the government-owned hospital to reduce vomiting and/or chloroquine-induced itching. Frequency of co-administration of chlorpheniramine with chloroquine was 75% in 1997, when the frequency of use of promethazine decreased to 25%.

**Table 6:** Relative frequency of antihistamines co-administered with chloroquine at government owned hospital.

	Promethazine		Chlorpheniramine		Cyproheptadine		Others	
	**N**	**F**	**N**	**F**	**N**	**F**	**N**	**F**

1983 N = 25	16	64%	4	16%	0	0	5	20%

1985 N = 25	14	56%	1	4%	0	0	10	40%

1987 N = 30	21	70%	1	3.3%	0	0	8	26.7%

1989 N = 23	15	65.2%	1	4.3%	0	0	7	30.4%

1992 N = 9	8	88.9%	1	11.1%	0	0	0	0

1995 N = 9	6	66.7%	2	22.2%	0	0	1	1.1%

1997 N = 4	1	25%	3	75%	0	0	0	0

N = No. of prescriptions evaluated

F = Frequency of prescription

### Trends of chloroquine sensitivity in south-west Nigeria

The prescription practices at the private hospital based on use of therapeutic or sub-therapeutic dosages as well as co-administration of anti-histamines with chloroquine were collated with the reported trends of chloroquine sensitivity in south-west Nigeria (Table 7). The reported clinical sensitivity of *Plasmodium falciparum *to chloroquine decreased from 100% in the pre-resistance years to 40% in the resistance dissemination years. During the period of dissemination of resistance there were corresponding decreases in the frequency of prescription of full course treatment regimen of chloroquine and co-administration of antihistamines from 93% to 56% and 74% to 51% respectively.

**Table 7:** Trends in chloroquine sensitivity profiles during periods of co-administration of antihistamines with chloroquine or use of sub-therapeutic dosage regimen in patients from a private hospital in Ibadan, south-west Nigeria

Year	Frequency of co-administration of antihistamine	Frequency of prescription of Full course regimen	Frequency of sub-therapeutic regimen	Reported chloroquine sensitivity
1983 (N = 289)	74.7%	93.1%	6.9%	100% [19,20]

1985 (N = 298)	80.0%	98.3%	1.7%	

1987 (N = 295)	84.4%	96.3%	3.7%	100% [21]

1989 (N = 220)	72.3%	97.3%	2.7%	

1992 (N = 258)	67.4%	78.3%	21.7%	85% [22]

1995 (N = 243)	48.6%	72.8%	27.2%	

1997 (N = 241)	51.9%	56.4%	43.6%	<40%([3]

## Discussion

The study was conducted in two hospitals, a government health care facility and a private health care facility. The record keeping at the private health care facility was excellent and permitted a retrospective study over the period chosen. Record-keeping at the government health care facility was quite different with categorization of certain records as secondary records and thus stored away with limited access. This practice did not permit full access to the records required for the retrospective study. Availability of modern medical record system has been identified as a key factor facilitating clinical audit [11], thus modernizing medical record systems in Nigeria will be valuable in development of clinical auditing. The private sector is a leading provider of malaria case management in many endemic countries [12], which was also evidenced in this study. The results from the study show that during the pre-resistance years chloroquine was the main drug used in the chemotherapy of malaria in both sectors of health care provision. It was administered as standard doses during this period and anti-histamines, especially promethazine, were routinely co-administered with chloroquine. The practice of prescribing sub-therapeutic doses of chloroquine became prominent between 1992 and 1997 which coincided with the emerging resistance and resistance dissemination phases and revealed deficiencies in the private sector in relation to poor adherence to National and WHO guidelines for malaria. Poor drug use practices such as the use of sub-therapeutic doses, are among factors that can lead to the emergence and spread of drug resistant strains of *Plasmodium falciparum *([7] and this was evident in this study. The government-owned hospital, which is also a tertiary institution, adhered to National and WHO guidelines for malaria treatment. The findings from this study are consistent with previous reports [13] that prescribers in government health facilities tend to adhere more to national treatment guidelines than private practitioners. These findings pose a huge challenge to national treatment policies which are aimed at improving health care at all levels.

Promethazine and other anti-histamines are routinely used as adjunct to chloroquine in the treatment of malaria in south-west Nigeria to reduce chloroquine-induced pruritus and/or vomiting. The decline in chloroquine efficacy in south-west Nigeria appears to coincide with the period of increased prescription of sub-therapeutic doses of chloroquine and decreased co-administration of anti-histamines with chloroquine. Drug pressure and exposure of parasites to sub-therapeutic concentrations of anti-malarial drugs represent positive inducers of drug resistance.

Although chlorpheniramine or promethazine are routinely used as adjunct to chloroquine in treatment of malaria, the role of this prescription practice in the slow emergence of drug resistant parasites in Nigeria is unknown. Anti-histamines, including chlorpheniramine, promethazine and cyproheptadine, have been shown to enhance chloroquine activity by reversal of chloroquine resistance in vitro and in vivo [14-16]. The efficacy of the combination of chloroquine and chlorpheniramine in the treatment of uncomplicated acute chloroquine resistant *P. falciparum *infection was confirmed in limited clinical studies in southwest Nigeria [4,5]. In the present study, there was an association between decreased frequency of use of promethazine and emergence of chloroquine resistance. Thus, inadvertent combination of chloroquine with anti-histamines over the years may have contributed to delay in emergence of resistance to chloroquine in south-west Nigeria. A recent study also described beneficial pharmacokinetic interactions between chlorpheniramine and chloroquine [17]. These observations essentially represent a valuable proof of the principle for clinical application of the reversal of resistance phenomenon.

The Nigerian Government adopted the use of ACT in 2005 and the current guideline for treatment of uncomplicated falciparum malaria in Nigeria is artemether-lumefanthrine or artesunate-amodiaquine [18]. The results from the present studies confirm the influence of prescription of inappropriate doses of anti-malarial drug on dissemination of drug resistance falciparum malaria and the need to improve malaria treatment practices in Nigeria. In the resistance dissemination years, the records show that artemether was prescribed as monotherapy despite the fact that it was not one of the drugs recommended for management of malaria. Furthermore, in Nigeria, anti-malarial drugs are obtainable over the counter without prescription thus there is a potential for monotherapy with the artemisinins and the use of ACT in sub-therapeutic doses. As Nigeria implements the use of ACT, it becomes imperative that sub-standard doses of ACT are not used at any level of health care delivery. Since the private sector is a leading provider of malaria case management in many endemic countries [12] and responsible for treating over 50% of malaria cases in Nigeria, it is critical that the private sector in addition to government supported health care providers are targeted for training programmes in malaria case management.

## Conclusion

The study revealed that chloroquine was the most commonly prescribed anti-malarial drug throughout the phases studied. The prescription of substandard doses of chloroquine was more prevalent in the private hospital than the government hospital coinciding with the period of dissemination of resistance.

## Competing interests

The authors declare that they have no competing interests.

## Authors' contributions

GG was the principal investigator responsible for the study design, data collection, and analysis and manuscript preparation. CH, OO and AS provided valuable technical guidance and had critical input into the protocol and manuscript preparation. AG carried out field data collection and analysis. AO was the overall project leader who founded the idea of studying prescription practices and its relation to emergence of resistance and provided vital technical guidance in the development of the proposal, data collection, and analysis. All authors read and approved the final version of the manuscript.
